# Oral Biofilm and Its Connection to Alzheimer’s Disease

**DOI:** 10.7759/cureus.72841

**Published:** 2024-11-01

**Authors:** Gregori M Kurtzman, Robert A Horowitz, Richard Johnson, Zachariah Pedro

**Affiliations:** 1 General Dentistry and Implantology, General Dental Practice, Silver Spring, USA; 2 Periodontology, New York University College of Dentistry, New York, USA; 3 Internal Medicine, Retired, New York, USA; 4 Psychiatry, New York University, New York, USA

**Keywords:** alzheimer’s disease, coordinated care, dementia, oral biofilm, oral-gut connection

## Abstract

Dementia and Alzheimer’s disease are common occurrences in the population, affecting many patients. Recent research and studies have found a link between oral biofilm and the initiation of these conditions or the worsening of their presentation. Periodontal disease and the associated oral biofilm with its bacteria are often not considered by the medical community when treating these or their patients. Coordination of therapy with a dentist can improve the patient’s oral health. Decreasing bacteria in the oral biofilm allows the physician and dentist to provide coordinated total healthcare. Emphasis and education of the patient on the importance of maintaining good oral homecare and routine dental recall prophylaxis appointments to improve their systemic health and limit the progression and worsening of mental health conditions. This article discusses the connection between oral biofilm and systemic health, specifically Alzheimer’s disease, and how to improve those conditions through oral healthcare.

## Introduction and background

**Figure 1:**
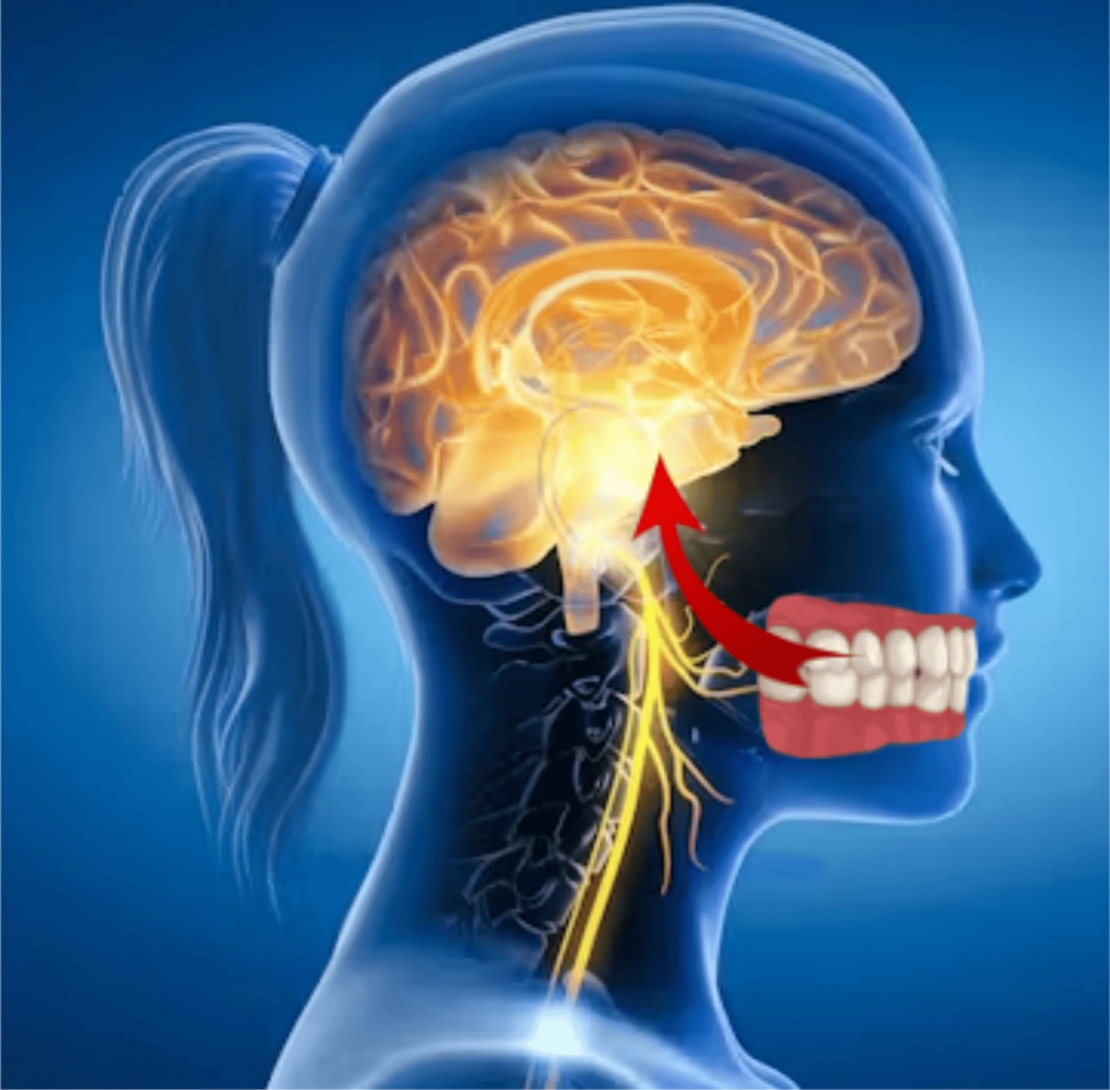
Connection between the oral environment and the brain Image Credit: Gregori M. Kurtzman (created using Adobe Photoshop without the use of artificial intelligence)

Physicians are in a unique position to access and treat their patients’ total health. As this is managed, they may be the first healthcare practitioner to identify the presence of or manage issues with these systemic health issues. Increasing evidence of either initiating systemic issues or complicating those medical conditions has been reported, linking oral biofilm and their associated bacteria to numerous systemic issues (Figure 1).

The patient’s health needs to be considered as a whole-body system that has connections originating in the oral cavity, having distant effects throughout the body. Thus, the dentist is part of the healthcare team identifying and treating oral biofilm that may be present. A “team approach” needs to be coordinated for those patients with systemic issues. This can help eliminate factors that may be causing or exacerbating the systemic issues or making those resistant to medical treatment. To maximize total health, coordination in healthcare needs to be a symbiosis between the physician and dentist to eliminate the oral biofilm and aid in the prevention of systemic disease. This will minimize these effects and aid in improving the patient’s overall health and quality of life. This article will focus on oral biofilm and its connection with Alzheimer’s disease and how improving oral care can limit the progression of the disease.

## Review

What is an oral biofilm?

Oral biofilm, formally referred to as dental plaque, due to its complex nature, has emerged in the literature. It has long been associated with periodontal disease and, to a lesser extent, dental caries. The disease process relates to the bacteria contained within the oral biofilm. The oral bacterium has long been ignored for any effects outside the oral cavity. Accumulating research has directly provided a link between the patients' oral health and the systemic diseases that may be present [1,2]. Two hundred possible connections between systemic diseases and oral health have been reported by the American Dental Association [3,4]. The accumulating evidence has linked periodontal disease and chronic oral inflammation to multiple health conditions, including cardiovascular disease, renal issues, diabetes, osteoporosis, pulmonary disorders, Alzheimer’s disease, and other systemic conditions [5]. With the reported research in mind, oral biofilm has been recognized as a more complex environment than previously understood [6,7].

Oral biofilm consists of a microorganism community found on the tooth surface or within the gingival sulcus (periodontal pocket), which is embedded in a polymer matrix of host and bacterial origin. Over 700 different bacterial species naturally reside in the mouth, with most being considered innocuous. Some of those microorganisms have been identified as pathogenic. As bacterial numbers increase, those microorganisms quickly create an intricate network of protective layers (i.e., polymer matrix) with channels developing into a biofilm, which is the major cause of periodontal disease. Those oral biofilm bacteria being protected by that polymer matrix are less susceptible to antimicrobial agents, either locally or systemically administered. These microbial communities have been long demonstrated to display an enhanced pathogenic synergism with the other residing bacterial species [8,9]. The structure of the biofilm additionally may restrict the penetration of antimicrobial agents. On the contrary, bacteria growing on a surface (planktonic) are susceptible to antimicrobial agents [10]. Bacteria in the oral biofilm have been reported to provide drug resistance to antibiotics and other medicaments, making it difficult to chemically control those microorganisms [11,12].

Through aggregation, the bacteria in the biofilm work together as a community, producing specific proteins and enzymes by way of quorum sensing, utilizing oral fluids as the vector for transmission [13,14]. Bacteria in these oral environments have evolved as part of multispecies biofilms requiring interaction with other bacterial species to grow, forming complex bio-environments [15,16].

Quorum sensing is a cell-to-cell communication mechanism that synchronizes gene expression in biofilms in response to the density of the cell population, giving oral biofilm bacteria the ability to regulate numerous processes [17,18]. That includes the secretion of specific enzymes to activate or deactivate the genes of other bacteria. Those bacterial byproducts provoke a host immune response, recruiting white blood cells (WBCs) to the site to kill the invading bacteria, resulting in localized inflammation in the surrounding gingiva. The bacteria using quorum sensing have the ability to confuse those defending WBC chemotactically, releasing chemicals into the local environment and rendering the immune response ineffective. WBCs have a three-day life cycle. If they do not engulf a microorganism and destroy it within that timeframe, the WBCs lyse and die [19]. Components within the WBC that were intended to kill bacteria are now available to damage the very tissue they were meant to protect. This process contributes to gingival inflammation, progressing to periodontal bone loss and a deepening of the periodontal pockets [20-22].

The biofilm bacteria are a diverse community, with variations in the many species being detected. In the same patient, the biome can be different from site to site. Once formed, the species composition of the biofilm has a degree of stability at a site among component species due to a balance between synergism and antagonism [23,24]. The oral biofilm in periodontal pockets is most likely to be seen in its mature state. As the periodontal pockets deepen and are less accessible to the patient’s home care, these areas provide protection from removal by the patient [25-27]. As that biofilm matures, the microbial composition changes from one that is primarily gram-positive and streptococcus-rich to a structure filled with gram-negative anaerobes [28,29].

Initially, the biofilm biome consists of predominately gram-positive cocci bacteria (*Streptococcus mutans*, *Streptococcus oralis*, *Streptococcus sanguis*, *Streptococcus mitis*, *Staphylococcus epidermidis*, and *Rothia dentocariosa*). That is followed by some gram-positive rods and filaments (*Actinomyces gerencseriae*, *Actinomyces viscosus*, *Actinomyces israelis, *and *Corynebacterium species*), with a very small number of gram-negative cocci. *Veillonella parvula* and *Neisseria species* comprise some of the gram-negative cocci and are aerobes or facultative aerobes. The early biofilm is able to withstand frequent mechanisms of oral bacterial removal, such as swallowing, chewing, and salivary fluid flow. Additionally, early bacterial colonizers are able to survive high oxygen concentrations in the oral cavity. This initial biofilm, which is always present orally, forms immediately after cleaning via toothbrushing or professional dental prophylaxis.

Later bacterial colonizers undergo co-adhesion to the initial biofilm, which involves specific interactions between bacterial receptors that increase the biofilm volume. This results in a more complex and diverse environment, with those diverse bacterial species creating synergistic and antagonistic biochemical interactions among the colony inhabitants. This metabolically contributes to bacteria that are physically near them. When obligate aerobes and anaerobes are involved in co-adhesion, those interactions aid in the survival of the anaerobic bacteria in the oxygen-rich oral environment. Those bacteria continue dividing until a three-dimensional mixed-culture biofilm has formed that is specially and functionally organized. Polymer matrix production by the bacterial inhabitants of the biome leads to the development of an extracellular matrix, a key structural aspect of the biofilm. This matrix offers protection to the microbial inhabitants from external factors. As that biofilm thickens as it matures, anaerobic bacteria can live deeper within the biofilm, further protecting them from the oxygen-rich oral environment.

Patients with oral biofilms do not need to present with gingival bleeding to have systemic issues related to the microorganisms in the biofilm. Identification of the presence intraorally is directed by the dentist to identify the presence or absence of periodontal disease (deepening pockets). Residual biofilm is initially identified by the presence or absence of deepening periodontal pockets. The presence and severity of periodontal disease vary according to factors including the individual patient’s immunological response to the bacteria in the biofilm. Some patients present with typical signs of periodontal disease, such as bleeding on probing or brushing and gingival inflammation. However, other patients do not present with these typical signs or symptoms.

Harmful strains of bacteria in the oral biofilm may enter the bloodstream during the inflammatory response, traveling to other areas of the body. Once there, they can exert systemic effects that have been linked to numerous diseases. Increasing evidence has been reported that indicates patients with periodontal disease have a much higher risk of developing cardiovascular and other systemic issues than those individuals who take preventive measures to eliminate and control the biofilm in their mouths [30,31].

Oral-Gut Connection

The oral environment and gut have the two largest microbial environments, which are interconnected, playing a major role in microbiome-associated diseases [32]. That oral-gut connection may be a route linking periodontal and systemic diseases, with strong correlations being reported between oral and fecal bacterial species [33]. Evidence suggests that periodontal-associated pathogens may translocate to distant sites, eliciting severe local and systemic pathologies. Current modes of treatment to reverse microbial dysbiosis include fecal microbiota transplantation, probiotics, prebiotics, and synbiotics through the introduction of health-related bacterial species and substrates [34].

Oral pathogens may disseminate to distant organs via localized oral blood circulation or be swallowed and then passed through the gastrointestinal tract, entering the systemic circulation. Once those oral pathogens reach an organ, they can modify the immune response, stimulating the release of inflammatory mediators, which then can result in systemic disease [35,36]. The precise role of those oral microbes in systemic organs, including the gut, remains elusive [37]. Dissemination of those oral microbes to the gut may exacerbate various gastrointestinal diseases, including irritable bowel syndrome, inflammatory bowel disease, and colorectal cancer. Further research is needed to confirm the oral-gut connection and its effect on systemic health related to the bacteria and their byproducts found in these interconnected environments. Daily use of probiotics has been shown to improve conditions and aid in decreasing the bacteria associated with systemic issues originating in the gut [38]. Other effects noted have been associated with cognitive changes that may develop into Alzheimer’s disease [39].

Alzheimer’s Disease

Alzheimer`s disease is a prevalent cause of dementia and is often assumed to be caused by an aggregation of extracellular beta-amyloid and intracellular tau protein [40,41]. This has been supported by a recent study showing reduced brain amyloid levels and reduced cognitive decline under treatment with a beta-amyloid-binding antibody [42].

Various microorganisms have been detected in the cerebrospinal fluid and brains of AD patients, including *Porphyromonas gingivalis* and *Spirochaetes*. Those bacteria are also found in the oral cavity under normal conditions and are often affected by or related to multiple dental pathologies, including periodontal disease and caries. Additionally, recent studies have suggested that periodontal disease and alterations in the oral microbiome may be associated with cognitive decline and Alzheimer's disease development [43]. Bacterial agents, including periodontal pathogens, have been increasingly reported to be important in the pathology of Alzheimer's disease [44]. The associated bacteria include *Porphyromonas gingivitis*, *Prevotella melaninogenica*, *Campylobacter rectus*, *Prevotella nigrescens*, *Fusobacterium nucleatum*, *Streptococcus intermedius*, *Capnocylophaga ochracea*, and *Prevotella melaninogenica*. Increasing evidence is being reported of an association between periodontal pathogens and Alzheimer’s disease, especially in the older population [45,46]. Those periodontal pathogens and the associated subsequent chronic inflammatory responses have significant implications for the development of Alzheimer's disease. Reported data has demonstrated that periodontitis is associated with an increase in cognitive decline in Alzheimer's disease, which can be mediated through effects on systemic inflammation [47]. The exact mechanism of periodontal pathology and its involvement in the pathogenicity of Alzheimer's disease, however, is not currently known. Yet certain oral biofilm bacteria can alter the host immune response in Alzheimer's disease [48,49]. There appears to be a risk factor between patients who have Alzheimer’s disease and those with periodontal disease. Addressing the oral factors may stop or slow the progression of the neurological condition [50].

Management of periodontal disease and its inflammatory mediators should slow the progression of cognitive decline and extend the patient’s quality of life. Specifically, *Porphyromonas gingivalis* stimulates host immune cells and releases cytokines, lysosomal enzymes, nitric oxide, and reactive oxygen species, which leads to cell damage, apoptosis, and inflammation. Periodontal disease through the systemic inflammation it causes related to the associated bacteria leads to some problems. The progression of mild cognitive impairment can result from the production and aggregation of beta-amyloid and tau proteins in the brains of elderly patients [51].

How can oral biofilms be managed?

Biofilm management involves treatment by the dentist to identify periodontal disease, manage the disease process, and return the periodontal tissue to a more normal, healthy state. However, it also involves improved patient homecare to keep biofilm levels down and prevent periodontal disease resurgence and the reported associated systemic effects.

Mechanical debridement by homecare of the pocket is unable to remove all of the biofilms as toothbrushes are poorly effective, more than 4 mm subgingivally, under the best efforts of the patient. Re-growth of the biofilm occurs within three hours, resulting in a fourfold (400%) increase in biofilm mass [52]. Homecare, regardless of how diligent the patient tries to be, is compromised as the toothbrush bristles are unable to extend more than 3-4 mm into the pocket and are unable to mechanically contact the biofilm located at deeper depths [53]. Use of dental floss only reaches 1-2 mm into the pocket, preventing mechanical removal of the oral biofilm. A similar problem was observed with oral irrigators (i.e., Waterpik) and similar devices, as they do not allow irrigation to the bottom of the pockets, and most patients are not diligent in their daily use. The sulcular environment is difficult for most patients to reach with brushing and flossing, which makes it impossible to control oral biofilms by mechanical means alone as the bacteria grow and replicate rapidly. Post-cleaning biofilm redevelopment is more rapid and complex, exceeding pre-cleaning levels within two days [54,55].

Chlorhexidine oral rinses have been reported to have an effect on young biofilms, but the bacteria in mature biofilms and nutrient-limited biofilms have been shown to be more resistant to their effects [56,57]. Additionally, chlorhexidine has been linked to negative effects on fibroblasts and other cells in the soft tissue of the sulcus [58]. On the other hand, chlorine dioxide has been demonstrated as an effective oral rinse that functionally debrides the biofilm slime matrix and bacterial cell walls, essentially peeling the biofilm back layer by layer. Chlorine dioxide has not been reported to have any negative effects on fibroblasts or other host tissue cells either dentally or systemically and is safe for daily use in patients. Stabilized chlorine dioxide (Cloralstan) has been reported in the literature to reduce both plaque and gingival indices and bacterial counts in the oral cavity, similar to other routinely used oral rinses [59]. The solution has shown high safety and efficacy, with concentrations of up to 40 ppm in drinking water reported to not show any toxicity in subchronic oral toxicity tests [60,61]. The lack of cytotoxicity to human cells and selective toxicity to bacteria appears to be related to the cell membrane structure. The best benefit when using chlorine dioxide oral rinses is to brush the teeth and gums with it following swishing, prior to spitting it out to aid in getting the rinse into areas that swishing along can not reach (between the teeth and into the gingival pockets). Oral rinsing on its own is unable to reach more than 1 mm subgingivally. Utilization of oral rinses in an oral irrigator allows for deep flushing of the periodontal pockets to increase biofilm removal [62].

Partial and full dentures are prone to accumulating oral biofilm. Many of these patients are often older, physically or in other ways compromised, and need to have their caretakers daily clean those appliances when they are not capable. Cleaning them with a toothbrush with a chlorine dioxide oral rinse aids in eliminating the oral biofilm. This also aids in preventing aspirating that biofilm, which can introduce it to the pulmonary system and decrease the potential for pulmonary infections that are common in the senior population.

As bacteria embedded in the biofilm are up to 1000-fold more resistant to antibiotics than planktonic bacteria [63], the use of antibiotics either systemically or in oral rinses and site application has been shown to be unable to eliminate or manage the biofilm bacteria adequately [64]. This has implications both with natural teeth and periodontal issues developing around dental implants, leading to peri-implantitis [65].

Photobiomodulation (PBM), also referred to as low-level laser therapy, has demonstrated benefits with temporomandibular joint issues, clenching and its related muscle soreness, as well as antibacterial application to oral biofilms that are not easily accessible subgingivally by the patient [66,67]. Utilization of PBM in the red (600-700 nm) and near-infrared wavelengths (780-1100 nm) intraorally has the ability to penetrate the gingival tissue to irradiate the oral biofilm in the gingival sulcus. Additionally, bacterial elimination of *Streptococcus mutans* in the biofilm has demonstrated a decrease in caries, a benefit to all patients, but specifically those elderly patients who, with decreases in mental health related to dementia, are not able to maintain routine oral homecare [68].

A study reported a significant reduction in bacteria when PBM was applied to the skin on a routine basis [69]. Usage of PBM applied intraorally in treating oral diseases such as tooth caries, pulpitis, periodontal diseases, peri-implantitis, and oral candidiasis permits an easy-to-utilize method to supplement standard oral homecare (brushing, flossing, and use of oral irrigator) [70]. Additionally, use in the management of lichen planus [71] and oral mucositis [72], which are common afflictions seen in the aging population, is an added benefit to prevent those conditions and manage them when they present in the patient.

**Figure 2:**
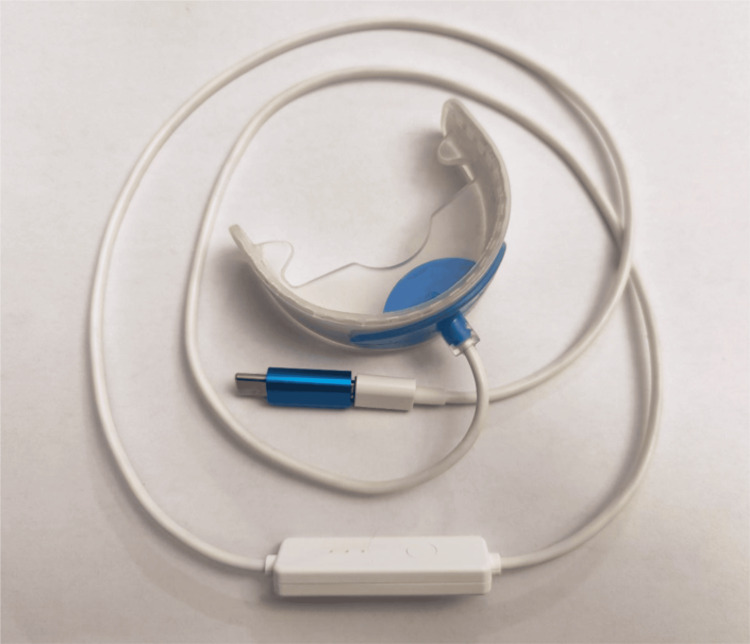
Accelite intraoral device and power cord that connects to a cell phone Image Credit: Gregori M. Kurtzman

**Figure 3:**
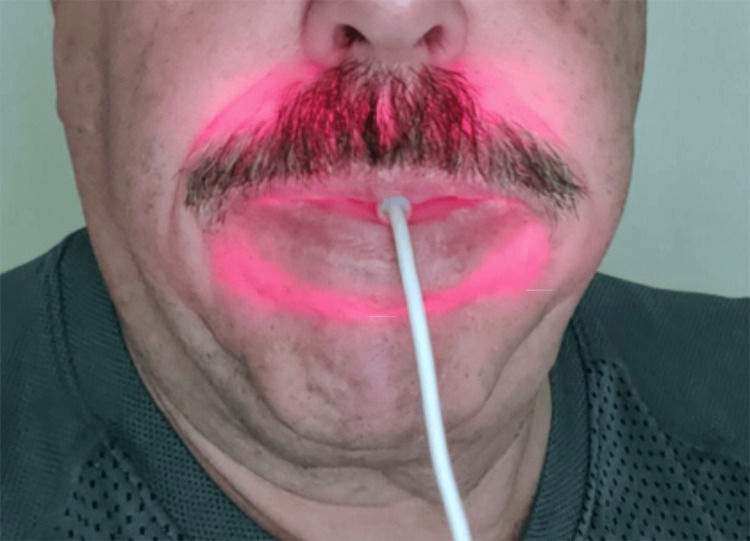
Accelite device placed intraorally and activated demonstrating illumination throughout the oral environment Image Credit: Gregori M. Kurtzman

Patient compliance centers around minimal time involved in utilizing it and its ease of use. The Accelite device (Accelite, Palm Beach Gardens, FL) is an intraoral PBM device that is placed into the mouth and the patient lightly closes on it (Figures 2-3). If the patient is edentulous, this should be done with the dental appliances (dentures) out of the mouth so that the appliance is not blocking penetration of the light into the tissue. The device is activated and utilized until the timer turns off the light after a five-minute irradiation period. It is recommended this be used once daily, but utilization more often has no negative effects, and the authors recommend using it twice daily (morning and evening) so that it becomes routine.

## Conclusions

Oral biofilm is recognized as a much more complex material that functions through the coordination of bacteria within a protective matrix. Extensive research has demonstrated that oral biofilms causing periodontal disease have distant systemic effects and have been connected to numerous medical conditions, which are supported in the literature. The absence of oral bleeding on probing or when brushing does not rule out the presence of a periodontal condition or oral biofilm in the gingival sulcus. A dental evaluation is recommended to eliminate any potential systemic effects from oral biofilm present, and instructions must be given to the patient to improve their daily homecare.

Alzheimer’s disease as well as other systemic conditions have been connected to bacteria found in oral biofilm. Periodontal treatment is evolving to be a major component of full-body medical care. Controlling the associated biofilm yields better overall systemic health. Improving daily homecare as part of their routine can aid in the elimination of the issues that have been associated with oral biofilm helping total healthcare and any complications it can have on systemic disease. Additionally, encouraging patients with systemic health issues to get routine dental care and improve their oral health can have a positive effect on their total health. With regard to early signs of dementia and those patients with Alzheimer’s, improvement in their oral health may slow down the advancement of those mental health conditions. It is advised to recommend a dental evaluation as a part of overall treatment to aid in improving the patient’s quality of life.
